# The emerging role of effector functions exerted by tissue-resident memory T cells

**DOI:** 10.1093/oxfimm/iqae006

**Published:** 2024-06-14

**Authors:** Norifumi Iijima

**Affiliations:** Center for Drug Design Research, National Institutes of Biomedical Innovation, Health and Nutrition (NIBN), Ibaraki, Osaka, Japan

**Keywords:** Tissue-resident, memory T cells, reactivation, effector function, cognate antigen recognition, non-cognate antigen recognition, infection, autoimmune diseases, allergic diseases, tumor progression

## Abstract

The magnitude of the effector functions of memory T cells determines the consequences of the protection against invading pathogens and tumor development or the pathogenesis of autoimmune and allergic diseases. Tissue-resident memory T cells (T_RM_ cells) are unique T-cell populations that persist in tissues for long periods awaiting re-encounter with their cognate antigen. Although T_RM_ cell reactivation primarily requires the presentation of cognate antigens, recent evidence has shown that, in addition to the conventional concept, T_RM_ cells can be reactivated without the presentation of cognate antigens. Non-cognate T_RM_ cell activation is triggered by cross-reactive antigens or by several combinations of cytokines, including interleukin (IL)-2, IL-7, IL-12, IL-15 and IL-18. The activation mode of T_RM_ cells reinforces their cytotoxic activity and promotes the secretion of effector cytokines (such as interferon-gamma and tumor necrosis factor-alpha). This review highlights the key features of T_RM_ cell maintenance and reactivation and discusses the importance of effector functions that T_RM_ cells exert upon being presented with cognate and/or non-cognate antigens, as well as cytokines secreted by T_RM_ and non-T_RM_ cells within the tissue microenvironment.

## Introduction

Secondary lymphoid organs (SLOs) are well-organized tissues for immunocompetent cells to initiate differentiation into effector cells, to combat against blood-borne pathogens, and transport antigens from the tissues. SLOs includes the spleen, lymph nodes (LNs), Peyer’s patches, tonsils, mucosal-associated lymphoid tissues, and adenoids. The distribution of lymphocytes, mainly T and B cells, in SLOs optimizes their interaction with foreign antigens that drain to the SLOs via the blood or lymphatics [1]. To this end, antigen-captured dendritic cells (DCs) from tissues are required to migrate into SLOs, or antigen-loaded SLO-resident DCs must enter T- or B-cell follicles in SLOs to maximize the activation of lymphocytes in SLOs [1]. In addition to immune cells, non-hematopoietic stromal cells construct microanatomical niches in SLOs to maximize immune responses and the differentiation into effector cells. This implies that all the immune cells and stromal cells are appropriately prearranged for lymphocyte activation and differentiation within the framework of the small compartments of SLOs [2, 3].

In contrast to SLOs, peripheral tissues containing barrier tissues (eye, mouth, lung, skin, stomach/intestine, and genitourinary tract), and non-barrier tissues (brain, liver, and kidney) are not sophisticated to educate antigen-unexperienced cells to differentiate into effector cells, except for tertiary lymphoid organs [4, 5]. The human body comprises various cells, tissues, and organs, each playing a specific role in maintaining stability and life. The internal environment of the body is maintained at a constant level despite various internal and external stimuli. However, owing to some causes (external factors, such as stress or infection), the balance of the internal environment of the body is disrupted, and health cannot be maintained. Therefore, major illnesses can be avoided by quickly stopping abnormalities at the forefront of internal causes and infections. Focusing on frontline defense in the body, it has been known for more than three decades that many lymphocytes are localized in peripheral tissues [6, 7]. In particular, the number of antigen-experienced memory T cells (T_M_ cells) is approximately 1–3 × 10^10^ cells in the skin, lung, and intestinal tissues, compared with the number of 0.5 × 10^10^ cells or 20 × 10^10^ cells in the blood or SLOs, respectively [7–10]. Furthermore, the functional and phenotypic characteristics of T_M_ cells in each tissue are entirely distinct from those in the blood or SLOs, exhibiting tissue-specific gene signatures [11–13].

Most T cells in human blood are naïve T cells that are not sensitized to antigens and express CD45_RA_ [14]. However, instead of CD45_RA_, CD45RO^+^ T cells have also been detected in the blood. These T cells are antigen-experienced T cells. Early in the immune response, CD45RA^+^ T cells are rapidly converted into CD45RO^+^ T cells, which are effector T cells (T_E_ cells) [15]. Furthermore, a group of CD45RO^+^ cells was found to be maintained for long periods as T_M_ cells, with two subsets in humans, as defined in 1991 [16]: effector memory T cells (T_EM_ cells) and central memory T cells (T_CM_ cells), each with predicted distinct phenotypes and functions based on the surface markers expressed on these T_M_ cells. T_CM_ cells patrol the blood and SLOs via the chemokine receptors CD62L (L-selectin) and C-C chemokine receptor (CCR)7. When stimulated, T_CM_ cells have a strong proliferative capacity and can differentiate into short-lived effector cells and T_EM_ cells [17].

In contrast, CCR7^−^ T_EM_ cells do not exhibit such molecular mechanisms and express distinct cell surface markers, including cutaneous lymphocyte-associated antigen (CLA), C-X-C chemokine receptor (CXCR)3, and CCR5 [16]. Therefore, it was thought that all T_EM_ cells continuously migrated to the peripheral tissues and returned to the bloodstream, and it is now conceivable that T_EM_ cells can be divided into two groups: those cells that express homing molecules such as the chemokine receptor CX3CR1, and CX3CR1^dim^ cells in humans and mice [18, 19]. The subset that can migrate to peripheral tissues is the CX3CR1^dim ^T_M_ cells, which refers to T_PM_ cells, and CX3CR1^hi ^T_EM_ cells are thought to exert effector functions in systemic infections [19]. Both types of T_M_ cells respond quickly to stimuli and produce various effector molecules but have limited proliferative capacity.

Until approximately the year 2000, it had been conceivable that a small proportion of T_M_ cell subsets could migrate to peripheral tissues as T_EM_ cells. Moreover, it was also extremely difficult to detect a minor population of T_M_ cells using flow cytometry in peripheral tissues containing CD45- cells, such as epithelial cells, fibroblasts, and endothelial cells, compared with SLOs, which contain approximately 90% immune cells. For this reason, most studies have used immunohistochemical analyses to evaluate the distribution of multiple types of immune cells in tissues. Thus, prior to detailed analysis of T_M_ cells in tissues, many of the mechanisms of T_M_ cell differentiation from T_E_ cells in SLOs, such as the spleen and LNs in mice and peripheral blood in humans, have been elucidated using flow cytometry [20–22].

As methods have been developed to prepare single-cell suspensions from tissues using various enzymes, it has become clear that many antigen-presenting cells (APCs) and T_E_ cells are localized in a wide variety of tissues [10, 23–25]. In terms of T_M_ cells in tissues, Masopust *et al*. detected antigen-specific CD8^+^ T_M_ cells in various murine tissues at least 5 weeks after viral infection [26]. In addition, the frequency of CD8^+^ T_E_ cells was higher in tissues than in SLOs. Simultaneously, Hogan *et al*. reported similar findings for antigen-specific CD4^+^ T cells in the murine lungs [27]. CD43^hi^CD69^hi^CD44^hi^CD4^+^ T cells that accumulated in the bronchoalveolar lavage protected against secondary viral infection.

Almost two decades ago, Clark *et al*. published a pioneering report suggesting that far more T cells than expected are localized in human skin tissues and contribute significantly to the defense against pathogens and tumors [8]. Many CLA^+^ and CCR4^+^ T_M_ cells have been isolated from human skin tissue, using the skin explant culture system [10]. Furthermore the migration of these T_M_ cells out of skin explants was controlled by chemoattractants, highlighting the importance of T-cell-mediated immunity in the skin [10]. These findings predicted that the T_M_ cells localized in the skin could become resident cells.

In 2004, Klonowski *et al*. demonstrated the long-term retention of antigen-specific CD69^hi^CD8^+^ T cells in the brain and intestinal tissues following viral infection using the technique of parabiotic surgery, which shares blood circulation between two mice [28]. These results initially indicate the long-term maintenance of T_M_ cells in various tissues. Later, Gebhardt *et al*. demonstrated the presence of tissue-resident memory T cells (T_RM_ cells) in the skin and dorsal root ganglia after cutaneous herpes simplex virus (HSV) infection following skin surgery or dorsal root ganglia transplantation [29]. These results confirmed that T_M_ cells localized in tissues become resident cells.

T_RM_, T_EM_, and T_PM_ cells all have the propensity to enter tissues. Therefore, a detailed study of their precursors is essential for distinguishing them. In the early phase of primary infection or reinfection in mice, KLRG1^-^ZNF683/homolog of B-lymphocyte-induced maturation protein 1 (Blimp-1) in T cells (Hobit)^+^ T_RM_ cell precursors in peripheral blood may be derived from CX3CR1^dim ^T_E_ or CX3CR1^dim ^T_M_ cells [30–32]. However, in human peripheral blood, T_CM_ cells highly express CLA, which enters skin tissue. Therefore, T_CM_ cells also can differentiate into T_RM_ cells in skin tissues [33]. In this case, specific factors such as chemokines must push the button for T_CM_ cells to infiltrate peripheral tissues and differentiate into T_RM_ cells.

T_RM_ cell precursors can access peripheral tissues, but sphingosine-1-phosphate receptors (S1PR) 1 and 5 expression and the high sphingosine-1-phosphate gradient between tissues and lymphatic vessels cause them to return to murine circulation for continued surveillance [34]. Furthermore, CCR7 expression on T_EM_ cells in mice appears to be a switch for exit from the tissue into the afferent lymph [35, 36].

Over the past decade, numerous studies have been published on the phenotypic, functional, and molecular characteristics of T_M_ cells that localize to tissues for long periods; we refer to the reader to other reviews for details of reports on the differentiation mechanisms of T_RM_ cells [37, 38]. In this review, we discuss why some memory T-cell subsets are programmed to be retained in peripheral tissues for long periods. The most plausible possibility is that T_RM_ cells play a specific role in the frontline defense against invading pathogens. However, tissue-specific autoimmune diseases may be regulated by the state of T_RM_ cells that recognize their antigens. Therefore, understanding the mechanism of T_RM_ cell function and identifying transcription factors and cell surface receptors for tissue retention of T_RM_ cells, and their effector functions following reactivation of T_RM_ cells , e.g. by pathogen reinfection, will greatly contribute to the development of new therapeutic interventions. Among them, targeting the effector functions of T_RM_ cells is a strong strategy for such interventions. Herein, we outline the role of T_RM_ effector functions in infectious diseases, autoimmunity, and cancer and discuss potential strategies to target T_RM_ cells to prevent and treat human diseases.

## Cytolytic and non-cytolytic functions of T_RM_ cells

### Cytolytic functions of T_RM_ cells

Fundamental knowledge of cytotoxic cell function is based on the secretion of lytic granules carrying several cytotoxic proteins [perforin-1 (Prf1), granulysin, and proteolytically active granzymes (Gzms)] formed as a glycoprotein shell based on thrombospondin-1 and serglycin, named by supramolecular attack particles and multivesicular bodies containing Fas ligand (FasL) [39–41]. Cytotoxic T cells (CTLs) and natural killer (NK) cells can exert a cytolytic effector function to eliminate target cells, such as virus-infected or tumor cells [39].

Upon interaction of the T-cell receptor (TCR) with specific antigens presented on major histocompatibility (MHC) class I expressed on target cells, CD8^+^ CTLs release Prf1, Gzms, and FasL toward the target cells. Prf1 creates pores in the plasma membrane of the target cells, allowing Gzms to enter the cytoplasm. Subsequently, intracellular Gzms trigger numerous signaling cascades that lead to cell death. During these steps, CD8^+^ CTLs promote a more powerful effector function by sequentially introducing cytotoxic effector molecules into their lytic granules [42].

In contrast to CTLs, γδT cells exhibit more efficient cytotoxic function without MHC-restricted mechanism [43]. To eliminate target cells, γδT cells utilize the interaction of costimulatory molecules, such as NKG2D, which is an activating receptor expressed on cytotoxic cells in the absence of TCR recognition [43]. NKG2D recognizes NKG2D ligands, including the MHC class I polypeptide-related sequences A and B (MICA and MICB, respectively), which are expressed upon stress, damage, or cell transformation.

NKG2D is expressed by a subset of γδT cells, CD8^+^ CTLs, and NK cells. Compared with γδT cells and CD8^+^ CTL, NK cells do not express antigen-specific receptors, whereas NK cells recognize the downregulation of MHC class I expression or the upregulation of several NK receptors, such as NKG2D ligands on damaged cells to secrete lytic granules. Furthermore, NK cells engage death receptors on the target cells via tumor necrosis factor (TNF)-α, FasL, and TNF-related apoptosis-inducing ligand (TRAIL), whose killing is slower than that mediated by lytic granules. Similar to γδT cells, NK cells can rapidly secrete lytic granules upon the recognition of virus-infected cells or tumor cells [44].

In addition, some of innate lymphoid cells (ILCs) secrete cytotoxic granules. In humans, CD127^-^CD94^+^ NK cells are cytotoxic, whereas CD127^+^CD94^-^ ILC1, ILC2, ILC3, and lymphoid tissue inducer cells are not cytotoxic [45]. Recently identified CD127^+^CD94^+^ ILCs are analogous to conventional ILC3 in terms of phenotypic and functional features and gene signatures in mice [46]. However, this population carries lytic granules, including Gzms and granulysins and exhibits cytotoxicity in response to interleukin (IL)-12 treatment. Furthermore, an increase in CD127^+^CD94^+^ cytotoxic ILCs was observed in patients with Crohn’s diseases [47].

In peripheral tissues and internal organs, a subset of γδT cells, ILCs, NK cells, CD4^+^ T_M_ cells, and CD8^+^ T_M_ cells become a tissue-resident population [37, 48–50]. In a microenvironment that barely retains residual antigens, CD8^+^ T_RM_ cells dampen the transcriptional levels of cytolytic molecules, including Prf1 and Gzms, before reinfection [51], which is potentially mediated by tumor growth factor (TGF)-β signaling [52]. However, following reinfection with lymphocytic choriomeningitis virus (LCMV) in mice, perforin-mediated cytotoxicity of CD8^+^ T_RM_ cells contributes to viral clearance in brain tissues [53], suggesting that CD8^+^ T_RM_ cells preserve lytic granules that contain cytotoxic effector molecules and then rapidly secrete them following the reinfection. Recent evidence has demonstrated that CD8^+^ T_RM_ cells in the murine liver, small intestine and cervix maintain the expression of cytotoxic molecules (GzmB, GzmK, FasL and TRAIL) [54, 55], whereas a lower frequency of CD8^+^ T_RM_ and CD4^+^ T_RM_ cells, which maintain GzmB, was observed in the female reproductive tract following LCMV infection [56]. In addition, GzmC was detected inside skin CD8^+^ T_RM_ cells before reinfection with vaccinia virus (VACV) in mice, and an immediate increase in GzmC and other Gzms was induced following CD8^+^ T_RM_ reactivation [57].

Although both CTLs and NK cells carry lytic granules that contain cytotoxic molecules to kill target cells, NK cells trigger the death of CD4^+^ T cells, followed by limiting the CD8^+^ T-cell response instead of killing virus-infected cells in a murine model of LCMV infection, but not mouse cytomegalovirus (MCMV) infection [58, 59]. Similarly, NK cell depletion increases the number of liver-resident CD8^+^ T_RM_ cells in a murine model of hepatitis B virus (HBV) infection [60]. In addition, the mechanism of limiting the CD8^+^ T_RM_ response was mediated by the upregulation of programmed cell death ligand 1 (PDL1), but not other NK cell-related receptors in liver-resident NK cells. Therefore, cytokine-activated liver-resident NK cells can negatively affect the CD8^+^ T_RM_ response in mice and humans [60]. Collectively, T_RM_ cells play a central role in protecting against pathogen reinfection, suggesting that the recognition of virus-infected cells through MHC molecules with cognate antigens is a critical step in ensuring pathogen clearance.

Considering the mode of the cytotoxic granule secretion from cells, CTLs and NK cells exhibit effector functions with strikingly distinct dynamics of calcium influx regulation. NK cells require short-term contact with target cells to kill them, whereas CTLs need to establish long-term contact to stabilize their interaction with target cells [61]. Furthermore, CTLs eliminate target cells by providing repeated pulses of sublethal hits until the target cells trigger death [62], suggesting that CTLs require much more time for elimination. T_E_ cells exert the highest cytotoxic activity, which is correlated with the secretion of lytic granules, including cytotoxic effector molecules [63]. In contrast, both T_CM_ and T_EM_ cells exhibited low cytotoxicity. On the other hand, CD45RA^+^ T_EM_ cells (T_RMRA_ cells) have been demonstrated to express Prf-1 and GzmB and are poised to kill target cells without activation in humans [64]. By comparison, human lung CD103^+^CD8^+^ T_RM_ cells maintained a large amount of GzmB mRNA without retaining proteins [65]. Although the mechanism of the rapid secretion of lytic granules in T_RM_ cells remains unclear, CD8^+^ T_RM_ cells, but not CD8^+^ T_EM_ cells, trigger the exocytosis of cytotoxic granules within 4 h of activation with cognate antigens in mice [54], suggesting that the ability to exhibit immediate cytotoxic function in CD8^+^ T_RM_ cells is transcriptionally regulated by a specific mechanism.

The transcription factor Hobit controls Gzm B expression in CD8^+^ T_E_ cells in mice and humans [54]. In addition, Hobit contributes to the maintenance of GzmB and TRAIL production in CD8^+^ T_RM_ cells and CD69 expression on CD8^+^ T_RM_ cells in the murine liver and small intestine [30, 54] (Table1). Unlike these tissues, Blimp-1, rather than Hobit, is necessary for CD8^+^ T_RM_ establishment in murine respiratory tissues [66], and CD69^+^CD8^+^ T_RM_ cells maintain a high expression level of GzmB, suggesting that the cytotoxic activity of CD69^+^CD8^+^ T_RM_ cells is regulated in a Hobit-independent manner. In murine skin tissue, Hobit expression was significantly elevated in CD8^+^ T_RM_ cells, liver NKT, and NK cells [67]. Both Hobit and Blimp-1 affect the expression of CCR7, Krüppel-like factor 2 (KLF2), CD69, S1PR1, and cytotoxic molecules, including GzmB and TRAIL [67]. Furthermore, in humans, Hobit expression is higher in blood T_M_ cells than in brain CD8^+^ T_RM_ cells [68] and lung CD4^+^ T_RM_ cells [69]. With respect to Hobit^+^ T_M_ cells in the blood, Hobit^+^CD8^+^ T_E_ cells can differentiate into T_RM_ cells in the liver, kidney, and small intestine [31]. Therefore, the requirement of Hobit and Blimp-1 for T_RM_ cell differentiation likely depends on the microenvironment of peripheral tissues.

**Table 1.** Transcription factors involved in the regulation of T_RM_ differentiation and function in mice and humans

Type of T_RM_ cells	Species	Location of T_RM_ cells *in vivo* or state of T_RM_-like cells *in vitro*	Transcription factor (TF) expressed in T_RM_ cells	Molecules or function controlled by TF or their impact on T_RM_ differentiation/survival	References
CD8^ + ^T_RM_ cells	Human	Skin Differentiation of T_RM_-like CD8 ^+^ T cells *in vitro*	Runx2, Runx3	Contribute to cytolytic function (GzmA and GzmB) and upregulate CD49a expression during T_RM_ differentiation	[72, 73]
		Colon Differentiation of T_RM_-like CD8 ^+^ T cells *in vitro*	Ahr	Promote T_RM_ differentiation and upregulate GzmB	[74]
		Renal cell carcinoma	Bhlhe40	Potentially promote T_RM_ differentiation and cytokine production	[75]
		Differentiation of T_RM_-like CD8 ^+^ T cells *in vitro*	Bcl11b	Upregulate the expression of CD69, CD49a, CD56, CD161, CD117, and NCR1 Downregulate the expression of CD62L and CCR7	[76]
		Cervix	Hobit	Expression in T_RM_ cells is associated with GzmB and GNLY expression.	[55]
		Lung	Notch-1, RBPJ, JAG2, ZEB2	Contribute to T_RM_ survival	[65]
			Bhlhe40	Promote T_RM_ differentiation and cytokine production?	[75]
			NFATc1	Potentially contribute to cytolytic function	[77]
CD4^ +^ T_RM_ cells	Human	Lung	Hobit	Persistence of cytolytic function	[78]
			HOPX	Potentially upregulate GzmA and GzmB expression	[78]
CD103^ +^ CD4^ +^ T_RM_ cells	Human	Lung	*Hobit, PRDM1, BATF, IRF4, EGR2, RBPJ ZEB2* (mRNA levels)	Contribute to T_RM_ survival	[69]
CD4^ +^ T_RM_ (Th17) cells	Human		c-Maf	c-Maf^hi^IL-10 ^+ ^Th17 cells in the presence of IL-27 upregulate CD69, CXCR6 and CTLA4.	[79]
CD8 ^+^ T_RM_ cells	Mouse	Intestinal tissues	Hobit	Upregulate the expression of GzmB and TRAIL Downregulate the expression of CCR7, KLF2 and Tcf1	[54, 67]
			Id2, Id3	Upregulate the production of IFN-γ, TNF-α and CD107a	[80]
			Bcl11b (Upstream of Tcf1 and Blimp-1)	Upregulate the expression of Ahr and the production of IFN-γ	[76]
			Tcf1	Upregulate the expression of Id3 and the production of IFN-γ and TNF-α Downregulate the expression of Blimp-1	[80]
			Ahr	Promote T_RM_ differentiation and upregulate GzmB	[74]
			Runx3	Promote T_RM_ differentiation and upregulate GzmB	[70]
		Lung	Bhlhe40	Promote T_RM_ differentiation and upregulate the production of IFN-γ, TNF-α and CD107a	[75]
			Blimp-1	Promote T_RM_ differentiation and upregulate CD69, CXCR6, and CD103	[66]
			NFATc1	Potentially contribute to cytolytic function	[77]
			EGR2	Control Notch-1 expression for T_RM_ differentiation	[81]
			Runx3	Promote T_RM_ differentiation and homeostasis	[70]
CD8 ^+^ T_RM_ cells	Mouse	Skin	Runx3	Promote T_RM_ differentiation, residency, and upregulation of PD-1, Tim-3, and GzmB	[70, 71]
			T-bet (Reduced expression)	Contribute to IL-15-mediated survival	[82]
CD8 ^+^ T_RM_ (T_RM_1) cells			Hobit, Blimp-1	Promote T_RM_ differentiation and upregulate CD103, CD69, CD49a and GzmB	[67, 83]
			T-bet	Promote CD8^ + ^T_RM_1 differentiation	[83]
CD8 ^+^ T_RM_ (T_RM_17) cells			c-Maf	Promote T_RM_ survival and upregulate IL-7R and ICOS expression	[83]
			Rorγt	Promote CD8^ +^ T_RM_17 differentiation	[83]
CD8 ^+^ T_RM_ cells		Liver	Hobit	Upregulate GzmB and TRAIL	[54]
		Kidney	Runx3, Hobit, Blimp-1	Promote T_RM_ differentiation	[67, 70]
		Salivary gland	Runx3	Promote T_RM_ differentiation	[70]
CD4^ + ^T_RM_ (Th1) cells	Mouse	Colon	Hobit	Promote T_RM_ differentiation and potentially contribute to cytolytic function	[84]
			Blimp-1	Promote T_RM_ differentiation and potentially contribute to cytolytic function	[84]
CD4^ + ^T_RM_ (Th1) cells		Lung	T-bet	Downregulate CD103 and CXCR3 expression	[85]
CD4^ +^ T_RM_ (Th2) cells			GATA3 Hobit↓Blimp-1↓RUNX3↓	Contribute to produce IL-5 and IL-13	[86]
CD4 ^+^ T_RM_ (T_RH_) cells			Bcl6	Contribute to produce IL-21	[87]
CD4 ^+ ^T_RM_ (T_RH_) cells			Bhlhe40	Potentially contribute to IFN-γ production	[88]
CD103 ^+^ CD4^ +^ T_RM_ cells			Notch-1, RBPJ	Necessary for T_RM_ cells to survive	[69]

Runx3 is highly expressed in CD8^+^ T_RM_ cells in the small intestine, salivary gland, skin and kidney of mice (Table 1) [70, 71]. Depletion of Runx3 in CD8^+^ T_RM_ cells clearly demonstrate that this transcription factor is crucial for the long-term maintenance of CD8^+^ T_RM_ cells [70] (Table 1). Furthermore, overexpression of Runx3 in CD8^+^ T_E_ cells increase GzmB expression, suggesting that CD8^+^ T_RM_ cells require Runx3 for GzmB production during differentiation. In the skin of mice, Runx3 is likely to promote TGF-β responsiveness in CD8^+^ T_RM_ cells for long-term maintenance [71] (Table 1).

As another transcription factor related to cytotoxic effector functions, NFATc1 regulates cytoskeletal reorganization, CD103 expression and cytotoxic organelle polarization in murine CD8^+^ T_E_ cells [77, 89]. Furthermore, NFATc1 is required for the differentiation of CD8^+^ T_RM_ cells [77]. Following the treatment with anti-PD-1 antibody, NFATc1 is upregulated in T_RM_ cells, possibly contributing to the exertion of cytolytic activity [77].

In the case of CD4^+^ T_M_ cells, Hobit is also highly expressed in human CD4^+^CD27^-^CD28^- ^T_E_ cells and T_RMRA_ cells, especially in human cytomegalovirus-specific CD4^+^ CTLs, but not in influenza A virus (IAV)- or Epstein-Barr virus-specific CD4^+^ T cells [90]. Similar to human CX3CR1^+^CD8^+^ T_EM_ cells, Hobit^+^CX3CR1^+^CD4^+^ CTLs maintain their cytotoxic effector functions for a long period. In addition, the frequency of CD103^+^CD4^+^ T_RM_ cells in human lung airway tissue was associated with the severity of asthma. CD103^+^CD4^+^ T_RM_ cells retain high levels of GzmA and GzmB [78] (Table 1), suggesting that the CD103^+^ population is cytolytic CD4^+^ T_RM_ cells. In murine colon tissue, Hobit is also upregulated in CD4^+^ T_RM_ cells but not in CD4^+^ T_EM_ cells, whereas the association of Hobit with cytotoxic activity in CD4^+^ T_RM_ cells remains unclear [84]. Another transcription factor related to the cytotoxic function of T_RM_ cells is the aryl hydrocarbon receptor (Ahr), which is controlled by Bcl11b and expressed in CD8^+^ T_RM_ cells. It affects the expression of Prf1 and GzmB in CD8^+^ T_RM_ cells in intestinal tissues of mice and humans (Table 1) [74, 76].

Collectively, a variety of immune cells carrying cytotoxic granules (T_RM_ cells, γδT cells, NK cells, and ILCs) are distributed in the microenvironment of peripheral tissues. These cells are thought to share roles depending on the microenvironmental conditions, and work to eliminate foreign substances and maintain homeostasis. However, excessive inflammation and other external factors may make it difficult to maintain the normal function of these cells.

### Non-cytolytic functions of T_RM_ cells

#### Cytokine production from T_RM_ cells

The critical function of T_RM_ cells is widely known to be a rapid effector function, including the secretion of cytotoxic effector molecules and cytokine production following TCR restimulation with cognate antigens in tissues. For instance, in the case of mucosal HSV-2 infection in mice, both CD4^+^ T_RM_ cells and CD8^+^ T_RM_ cells immediately produce IFN-γ within a day of reinfection, followed by elimination of infected cells to prevent viral dissemination into neuronal tissue [91, 92] and recruitment of memory B cells from the blood circulation [93], whereas reinfection with an irrelevant virus failed to produce IFN-γ from T_RM_ cells [91], suggesting that cognate antigen presentation is required for the rapid secretion of cytokines from T_RM_ cells. Consistent with this evidence, the upregulation of nuclear receptor subfamily 4 group A member 1, which is downstream of TCR signaling, in T_RM_ cells was observed in the lung tissues of mice on the day of reinfection with IAV [94].

Furthermore, the injection of cognate peptides into mucosal tissues where T_RM_ cells are localized, triggers the rapid secretion of IFN-γ, which triggers chemokine secretion and upregulation of vascular cell adhesion molecule 1 on endothelial cells, activation of innate immune cells, recruitment of memory B cells, and increased vascular permeability in tissues to reinforce protection against invading pathogens [95–99]. Therefore, cytokines secreted by T_RM_ cells can act on infected cells and have indirect effects that broadly enhance the protective function of other immune cells and non-immune cells in the microenvironment.

The transcription factor T-bet is the master regulator of IFN-γ and is sufficient for the induction of IFN-γ expression in CD4^+^ T_E_ cells [100]. On the other hand, IFN-γ secretion by CD8^+^ T_E_ cells is also mediated by Eomesodermin expression [101]. In T_RM_ cells, T-bet is expressed at lower levels than in T_E_ cells [85] and is also required for IL-15-mediated survival of CD103^+^CD8^+^ T_RM_ cells in the skin of mice (Table 1) [82]. In contrast, Bcl11b is also involved in the secretion of IFN-γ and TNF-α in CD8^+^ T_RM_ cells of intestinal tissues in mice [76]. Knockdown of Id2 and Id3 in CD8^+^ T_RM_ cells in the small intestine of mice increases the frequency of CD11b^+^ T-cell immunoglobulin and mucin domain 3 (Tim-3)^+^ subsets, whereas the subsets with cytokine-producing capacity (IFN-γ, TNF-α, and CD107a) are decreased [80].

Bhlhe40 appears to be involved in the survival and functionality of pulmonary CD8^+^ T_RM_ cells in mice and humans [75]. Bhlhe40 triggers the expression of multiple genes related to mitochondria, leading to oxidative phosphorylation and mitochondrial fitness [75]. Since Bhlhe40 controls both IL-10 and IFN-γ production in T_E_ cells [102, 103], it is likely that Bhlhe40 expressed in T_RM_ cells also regulates cytokine production, but the exact role of Bhlhe40 in T_RM_ cell function remains to be determined.

In contrast to IFN-γ production from T_RM_ cells, cognate antigen-dependent IL-5 and IL-13 production from GATA3^+^CD4^+^ T_RM_ cells in the lung parenchyma of mice augments eosinophil activation through up- and downregulation of CD11b and CD62L, respectively, leading to mucus metaplasia and airway hyper-responsiveness [86, 104, 105]. In contrast, circulating memory Th2 cells infiltrate the lung parenchyma and initiate perivascular inflammation to promote recruitment of eosinophils and CD4^+^ T cells [86, 106].

In addition to tissue-resident Th1 and Th2 cells, IL-21-producing programmed cell death protein 1 (PD1)^hi^FR4^hi^CD4^+ ^T cells become a resident population in the lungs of mice, called tissue-resident helper T cells (T_RH_ cells) [87, 88]. IL-21^+^CD4^+^ T_RH_ cells are localized in inducible bronchus–associated lymphoid tissue (iBALT) in mouse lung tissue and are required to inhibit cell death of a subset of CD8^+^ T_RM_ cells that require MHC class I recognition for their retention [88]. In particular, Bcl6 expression in CD4^+^ T_RH_ cells is critical for long-term maintenance through regulation of KLF2, IL-21, ICOS and IL-4 [87, 88].

#### Involvement of local antigens in the maintenance of long-term T_RM_ cell functions

To perform effector functions in tissues, T_RM_ cells must adapt to diverse microenvironments and establish long-term retention. It is widely accepted that CD103^+^CD8^+^ T_RM_ cells do not require local antigens for their maintenance [107], In particular, CD103 expression on CD8^+^ T_RM_ cells in the murine skin epidermis is independent of local antigen presentation [108]. In addition, the TCR responsiveness of CD103^+^CD8^+^ T_RM_ cells in the salivary glands, female reproductive tract (FRT), and small intestine of mice is dispensable for long maintenance periods [109].

In the early phase of infection, local antigens are not required to recruit T_E_ cells, but they augment the differentiation of T_RM_ cells from T_E_ cells in mouse tissues [110, 111]. In addition, the expression of CD69, CXCR6, and CD103 on T_RM_ cells is dependent on local antigen recognition [110–114]. Upregulating these typical T_RM_ cell markers requires interactions with local antigens during the early phase of T_RM_ cell differentiation. However, the need for the continuous recognition of local antigens to maintain the expression of these markers depends on tissue features. In contrast to the TCR-independent retention of CD8^+^ T_RM_ cells in the salivary gland, FRT, and small intestine of mice [109], persistent antigen retention in alveolar macrophages contributes to the long-term maintenance of CD8^+^ T_RM_ cells in murine lung tissues [115]. Furthermore, the persistence of local antigens from the IAV in the lungs of mice dictates the functional and phenotypic features of CD8^+^ T_RM_ cells. Following IAV infection, virus-derived nucleoprotein (NP) was detected at higher levels than polymerase acidic (PA) protein in the lung tissue several weeks after virus clearance [116, 117]. In line with antigen persistence, NP_366-374_-specific CD8^+^ T_RM_ cells, but not PA_224-233_-specific CD8^+^ T_RM_ cells, require MHC class I recognition and CD28 signaling for their maintenance [118]. In contrast, both of these CD8^+^ T_RM_ cells require TGFβ receptor (TGFβR) signaling for their retention [118].

Similar to CD103^+^CD8^+^ T_RM_ cells, the retention of CD103^+^CD4^+^ T_RM_ cells also requires TGF-βR signaling to maintain human epithelial layers [119]. In the human skin, approximately 50% of CD69^+^ T_M_ cells localized in the epidermis are CD103^+^CD4^+^ T cells, and in the dermis, nearly 40% of CD69^+^ T_M_ cells are CD103^-^CD4^+^ T cells. This is the first study to demonstrate that T_RM_ cells also exist *in vivo* in humans and that most of these CD52^-^CD69^+^ T_M_ cells are resistant to depletion of CD52^+^ circulating T cells following treatment with alemtuzumab, a monoclonal antibody that binds to CD52. This indicates that the remaining T_M_ cells in the skin are genuine T_RM_ cells [119]. In addition, T_RM_ cells in the skin preserve their ability to produce multiple cytokines, including IFN-γ, IL-17, granulocyte-macrophage colony-stimulating factor (GM-CSF), and IL-22.

In contrast, CD103^+^CD4^+^ T cells have also been detected in human peripheral blood and lymph [120]. Furthermore, CD103^+^CD4^+^ T_M_ cells in the blood are transcriptionally similar to CD103^+^CD4^+^ T_RM_ cells in the skin. These T_M_ cells, by contrast, do not express CD69. Therefore, the authors argued that CD69 downregulation from various CD103^+^CD4^+^ T_RM_ cells in the skin tends to recirculate into the blood circulation in a xenograft animal model [120]. However, human skin T_RM_ cells with the ability to recirculate into the blood circulation were IL-13^+^, IL-22^+^, or GM-CSF^+^ T_M_ cells, but not IFN-γ^+^, IL-17^+^, or IL-4^+^ T_M_ cells, suggesting that recirculatory capacity varies among T_M_ cell subsets. Another independent study reported that CD103^+^CLA^+^CD4^+^ T_M_ cells highly express GATA3 and produce IL-13, which is consistent with a previous study [121].

Compared with CD103^+^ T_RM_ cells, most CD103^- ^T_RM_ cells are localized to the lamina propria in mucosal tissues [122] or to the dermis in the skin [119]. The retention mechanism of CD103^-^CD8^+^ T_RM_ cells is likely to be distinct from that of CD103^-^CD8^+^ T_RM_ cells in terms of dependence on TGF-β signaling. CD103^-^CD8^+^ T_RM_ cells require CXCR3 to cluster with CXCR1^+^ APCs for long-term tissue maintenance [122]. Thus, the retention signal may be provided by CXCR1^+^ APCs, even though the stimulation with antigens is independent of CD103 expression.

The requirement for the interaction of MHC class II expressed on APCs for the retention of CD103^-^CD8^+^ T_RM_ cells remains unclear; however tissue-resident T_RH_ cells, but not PSGL1^+^CD4^+^ T_RM_ cells require MHC class II antigen presentation in murine lungs [87]. The largest population of T_RH_ cells is localized in B-cell clusters of iBALT in the lung, suggesting that MHC class II presentation on B cells is involved in the maintenance of T_RH_ cells.

The situation in the tumor microenvironment (TME) and organ transplantation differs from that of pathogen infection. In tumor-bearing tissues, continuous antigens derived from cancer cells modulate the features of T_RM_ cells [123]. Tumor-specific CD8^+^ T cells, a lineage of stem progenitors, infiltrate tumors and establish residency with the exhaustion phenotype. Thus, continuous stimulation with tumor antigens augments Tox expression in CD8^+^ T cells in humans [124]. However, exhausted T cells retain the expression of T_RM_ cell markers. Therefore, the molecular mechanisms of the retention of T_M_ cells are distinct from that of the exhaustion.

In contrast, the long-term persistence of T_RM_ cell populations following transplantation, as observed in the human skin, lungs, kidneys, and intestines, is associated with the development of graft-versus-host disease [72, 125–127, 128]. In mouse chronic kidney allograft rejection models, CD8^+^ T_RM_ cells are maintained through cognate antigen recognition and the IL-15 signaling pathway [129, 130]. In renal allografts, CD8^+^ T_RM_ cells are polyfunctional but not exhausted because they remain Tim-3 negative, although cognate antigens on MHC class I of CD11c^+^ APCs are chronically presented to these CD8^+^ T_RM_ cells [130]. Based on the findings of the tissue residency of T_RM_ cells in the model of tumor implantation and allograft transplantation, it is conceivable that chronic antigen presentation in tissues contributes to the maintenance of multifunctional T_RM_ cells unless the amount of cognate antigens is increased, such as the proliferation of cancer cells expressing MHC class I loaded with tumor antigens, or immune evasion strategies, such as release of immunosuppressive IL-10 or downregulation of MHC class I loaded with tumor antigens, are established in the tumor microenvironment [131].

## Protective roles of tissue-resident memory T cells

### Effector functions of T_RM_ cells to combat reinfection with viruses

For approximately two decades, T_M_ cells distributed in the peripheral tissues of mice and humans have been shown to produce effector cytokines (IFN-γ, TNF-α, GM-CSF, etc.) and cytotoxic granules (Prf1 and Gzms) [8, 26, 27, 132, 133]. In addition, local and mucosal immunization routes are superior in protecting against invading viral infection compared with systemic immunization [134–138]. Therefore, it has been suggested that T_M_ cells continuously infiltrate or persist as tissue residents in immunized tissues. A previous study demonstrated the possible retention of T_M_ cells in some peripheral tissues of mice, including the brain, peritoneal cavity, and intestinal lamina propria [28]. Using the parabiotic technique to share the blood circulation, transplantation or the prime and pull strategy, CD8^+^ T_RM_ cells have been experimentally identified, mainly in the epidermis and epithelial layer of mucosal tissues [29, 92, 139]. Furthermore, these cells have been shown to confer superior protection against rechallenge with LCMV [140], HSV-1 [29, 108], and VACV [139].

In contrast, most CD4^+^ T cells are distributed in the dermis (skin) or the lamina propria (mucosal tissues), suggesting that the mechanism for CD4^+^ T_RM_ cells for retention is different from that of CD8^+^ T_RM_ cells localized in the epidermis. In the case of genital herpes infection, attenuated HSV-2 immunization induces the formation of CD4^+^ T-cell clusters (MLC) beneath the lamina propria of genital tissues in mice [91, 141]. MLC formation is critical for rapid protection against genital HSV-2 rechallenge [91]. MLCs consist of many CD4^+^ T_RM_ cells and CD11b^+^ APCs, including macrophages and DCs, and a small number of CD8^+^ T_RM_ cells without PNAd^+^ high endothelial venules (HEVs). Furthermore, similar structures in which MLCs retain CD4^+^ T_RM_ cells are formed in murine skin dermis around hair follicles following HSV-1 skin infection [142].

In contrast to MLC formation, iBALT is generated in the lung tissue of mice following intranasal IAV infection [4]. The iBALT contains T-cell areas, including CD4^+^ T_RM_ cells, CD4^+^ T_RH_ cells, and CD8^+^ T_RM_ cells, and B-cell follicles, including CD21^+^ follicular DCs, with PNAd^+^ HEVs. Protection mediated by IFN-γ^+^CD4^+^ T_RM_ cells is superior to that mediated by circulating CD4^+^ T_M_ cells against IAV reinfection [85, 143]. In murine lung iBALT, IL-21-producing T_RH_ cells contribute to B cell immunity and local CD8^+^ T-cell help [87, 88]. The retention of PSGL1^+^ T_RM_ cells does not require antigen presentation, whereas FR4^+^ T_RH_ cells require cognate antigen presentation by CD20^+^ memory B cells for long-term maintenance [87].

Taken together, both CD4^+^ and CD8^+^ T_RM_ cells have been shown to contribute to protection against a variety of viral infections, including HSV-1, HSV-2, LCMV, MCMV, IAV, and VACV, particularly at the site of infection [29, 91, 139, 143].

### Effector functions of T_RM_ cells to combat reinfection with bacteria or fungi

Following *Listeria monocytogenes* (*L. monocytogenes*) reinfection, α4β7^+^CD8^+^ T_RM_ cells in the intestinal tissues of mice play a role in suppressing the bacterial load [144]. In lung *Yersinia pestis* infections, both CD4^+^ and CD8^+^ T_RM_ cells are required to confer protection against reinfection in an IFN-γ and IL-17-dependent manner [145]. CD4^+^ T_RM_ cells are also essential for protection against lung infection by the intracellular bacterium *M. tuberculosis* in mice [146]. CD4^+^ T cells in the lung parenchyma are superior to CD4^+^ T cells in the lung vasculature in protecting against *M. tuberculosis* infections. In addition, mucosal immunization provides CD4^+^ T_RM_ cell-mediated protection against pulmonary *M. tuberculosis* infection in mice [147–149].

*Salmonella enterica serovar* Typhimurium (*S.* typhi) is a virulent foodborne pathogen that infects humans and animals. *S.* typhi infection is initiated by the ingestion of contaminated food or water, which allows *S.* typhi to penetrate the intestinal epithelium and cause gastrointestinal disease. Immunization with a live vaccine strain of *S.* typhi generates IFN-γ-producing CD4^+^ T_RM_ cells that contribute to the protection against virulent *Salmonella* infection in mice [150].

In the case of urogenital infection, *Chlamydia trachomatis (CT)* and *Escherichia coli* (*E. coli*) are among the most common bacterial infections in humans. Following *CT* infection, lymphoid aggregates form beneath the genital epithelium, similar to MLC formation in the vaginal tissue of mice following attenuated HSV-2 immunization [151]. However, despite the presence of CD4^+^ T-cell clusters in the lamina propria of the genital tissues of mice, T_RM_ cells in these clusters do not appear to be required for protection against reinfection with *CT* [152]. In contrast to MLC formation which is composed of Th1 cells but not FoxP3^+^ regulatory T cells after attenuated HSV-2 immunization, intravaginal UV-inactivated *CT* immunization or *CT* infection in mice and humans results in a higher ratio of IL-10-producing FoxP3^+^ regulatory T cells and a lower ratio of IFN-γ-producing CD4^+^ T_RM_ cells in genital tissues [153–155], suggesting a lack of contribution to T_RM_ cell-mediated protection in genital tissues after *CT* secondary challenge. These data suggest that the quality of MLC formation following *CT* infection is of a distinct nature from that following attenuated HSV-2 immunization.

T_RM_ cells also accumulated in an antigen-dependent manner in the bladder tissue of mice following *E. coli* infection [111]. Both CD4^+^ and CD8^+^ T_RM_ cells protect against reinfection with *E. coli*, but the mechanism of T_RM_ cell-mediated inhibition of bacterial growth remains unclear.

Murine skin infection with *Candida albicans* results in the predominant development of IL-17-producing CD4^+^ T_RM_ cells that mediate protective immunity [156]. Intravital imaging shows two distinct populations of CD4^+^ T_RM_ cells with different migratory and functional properties. With respect to extracellular bacteria, CD4^+^ T_RM_ cells also mediate protection against *Streptococcus pneumoniae* colonization through the generation of IL-17-producing CD4^+^ T_RM_ cells [157].

### Effector functions of T_RM_ to combat reinfection with parasites

Similar to viral and bacterial infections, protection against parasite reinfection is associated with the functions of both CD4^+^ and CD8^+^ T_RM_ cells [158–160]. Although primary infection with parasites, including *Plasmodium berghei* ANKA, followed by chloroquine treatment generates CD4^+^ and CD8^+^ T_RM_ in the brain tissue of mice [161], it remains unclear whether these T_RM_ cells are protective against reinfection. To generate T_RM_ cells as a preventive vaccine strategy, prime and trap DNA vaccination, prime and target vaccination, and mRNA vaccines have been used to successfully generate T_RM_ cells with functional properties in the liver of mice [162–165].

Prime and target vaccinations aims to generate many T_RM_ cells in the liver [162]. Poly(lactic-co-glycolic acid)protein-loaded nanoparticles or viral vectors were administered intravenously. Cluster formation of antigen-specific CD8^+^ T_RM_ cells with hepatocytes is associated with protective immunity. In general, mRNA vaccination does not establish T_RM_ cells in tissues. However, combining mRNA immunization with αGarCer, an adjuvant, to activate NKT cells generated liver T_RM_ cells [164]. Furthermore, T_RM_ cells confer protection against sporozoite infection in mice. In contrast, lipid nanoparticle (LNP)-based mRNA vaccines trigger T_RM_ cells in the liver, contributing to protection against liver-stage malarial infection [165].

### Role of T_RM_ in tumor progression

Tumor development in peripheral tissues is a multistep process in which genetic alterations cause cells to divide, survive, and die in an uncontrolled manner. In contrast to the protective role of T_RM_ cells in infectious diseases, the requirement of T_RM_ cells for antitumor immunity has long been unclear. However, recent studies have demonstrated that T_RM_ cells play an essential role in antitumor immunity in various tumors using animal models [70, 123, 166–168]. In human studies, the abundance of T_RM_ cells in the TME is likely associated with the prognosis and beneficial clinical outcomes in patients with malignancies [169–173].

Diverse heterogeneity of memory T cells, including exhausted T cells (T_EX_) cells, follicular helper T cells (T_FH_), Th1 cells, TNFRSF9^+^ Treg cells, and T_RM_ cells, has been observed in the TME of humans [124, 174]. The heterogeneity of the T_EX_ cells was particularly noteworthy. Based on the results of a comprehensive gene analysis of T_EX_ and T_RM_ cells around the TME, it is highly likely that the suppression of T_EX_ cell differentiation and accumulation of T_RM_ cells play an important role in patient prognosis [124]. T_RM_ cells can prevent tumor develpment by constantly monitoring and eliminating transformed cells. However, once malignancy develops, tumor cells overwhelm the functions of T_RM_ cells and tumor-infiltrating T cells and deprive them of nutrition in the microenvironment, followed by the expansion of T_EX_ cell subsets. Tumor cells remove lipid uptake by CD103^+^ T_RM_ cells, which require lipid metabolism for survival. Consequently, CD103^+^ T_RM_ cells disappear around the human TME, whereas the survival of CD103^- ^T_RM_ cells is not affected by tumor growth. Thus, tumor growth deprives CD103^+^ T_RM_ cells of their lipid metabolism [175].

In addition to cancer immunosurveillance of T_RM_ cells in the TME, T_RM_ cells develop in distant tumor tissues, including LNs, prior to tumor metastasis and can protect against tumor dissemination in mice and humans [176–178]. Although the CXCR6-mediated retention of T_RM_ cells is required for inhibition of tumor progression, how T_RM_ cells block tumor metastasis remains unclear.

T_RM_ cell development is associated with tertiary lymphoid structures (TLSs) generated around the TME, which correlate with a positive prognosis in patients with cancer. In a variety of tumors, including gastric cancer and lung adenocarcinoma, the frequency of T_RM_ cells within TLSs in patients was significantly higher than that of T_RM_ cells outside TLSs and was positively correlated with patient outcomes [173, 179, 180]. In particular, mature TLSs comprise T-cell clusters containing T_N_, T_EM_, CXCL13^+^ T_FH_, and T_RM_ cells, with DC and CD20^+^ B-cell follicles organized by CD21^+^ follicular DCs and germinal center B cells. The ratio of mature TLSs is positively correlated with favorable prognosis in patients with cancer [5, 181–183]. In addition, T_RM_ cells outside the TLSs contribute to the inhibition of tumor progression [180].

Several studies have shown that treatment with an anti-PD-1 antibody increases T_RM_ cells in the TME by inducing proliferation and promoting the expression of fatty acid binding proteins 4 and 5 in T_RM_ cells, leading to an increase in lipid metabolism in T_RM_ cells, and preventing T_RM_ cell dysfunction by tumor growth in mice and humans [170, 175, 184, 185]. An efficient engineering method for inducing T_RM_ cells rather than accumulating T_EX_ cells in the TME, would be a promising new intervention for future cancer immunotherapy.

## Pathogenic roles of tissue-resident memory T cells

Although T_RM_ cells are critical for the rapid protection against infectious agents, uncontrolled T_RM_ cells may negatively affect the microenvironment. In addition to infectious diseases, CD4^+^ and CD8^+^ T_RM_ cells have been observed in various pathological conditions, including psoriasis, allergic asthma, inflammatory bowel disease (IBD), and other inflammation-related diseases in mice and humans [186–189].

### The condition in which protective T_RM_ cells generate pathogenic T_RM_ cells

Functionally dysregulated T_RM_ cells have been observed in several infectious and non-infectious diseases in both humans and animal models. As shown in various animal models and humans, T_RM_ cells express inhibitory molecules, such as PD-1, cytotoxic T-lymphocyte-associated protein 4 (CTLA4), and TIM-3, to varying degrees [68, 130, 190]. Despite expressing inhibitory molecules, T_RM_ cells exert their effector functions following pathogen invasion, suggesting that these T_RM_ cells are not terminally dysfunctional. However, although the levels of cytokine production (IFN-γ and TNF-α) in CD8^+^ T_RM_ cells in murine lungs increased after PDL1 blockade, the inhibition caused tissue injury and persistent fibrosis [118], suggesting that the inhibitory molecules expressed on T_RM_ cells control pathogenicity. In addition, the factor ‘age’ is involved in the dysfunction of CD8^+^ T_RM_ cells, contributing to chronic inflammation and fibrosis, regardless of the high expression of PD-1 on CD8^+^ T_RM_ cells in aged mice [191].

Although the following evidence does not show that protective T_RM_ cells directly transform into pathogenic T_RM_ cells, protective T_RM_ cells trigger the activation of bystander T_RM_ cells, causing tissue damage and inflammation. HBV-specific CD8^+^ T_RM_ cells in the human liver are critical for controlling chronic HBV infections [192]. However, in the liver microenvironment, along with HBV-specific CD8^+^ T_RM_ cells, non-specific CD8^+^ T_RM_ cells cause apoptosis in hepatoma cells in an MHC class I-independent manner [193], suggesting that chronic inflammation induced by the battle between HBV-specific CD8^+^ T_RM_ cells and HBV-infected cells leads to deleterious tissue damage. Likewise, non-specific CXCR6^+^CD8^+^ T_RM_ cells bearing similar functional features in non-alcoholic steatohepatitis (NASH) mice and patients with NASH kill hepatocytes through P2X7 purinergic receptors, but not through MHC class I recognition [194].

*Aspergillus fumigatus* colonizes the lungs of patients with chronic respiratory diseases and fibrosis. In these patients, *A. fumigatus*-specific CD4^+^ T cells in the lungs showed elevated IL-17A production [195]. Following repeated exposure to *A. fumigatus*-derived antigens, long-term resident CD4^+^ T cells trigger massive inflammation and fibrosis by secreting IL-4, IL-5, IL-13, IL-17A, and IFN-γ in animal models [196], suggesting that the chronic persistence of antigens in the lungs causes CD4^+^ T_RM_ cells to continue to secrete excessive amounts of cytokines. Thus, the inhibitory molecules on CD4^+^ T_RM_ cells may be dysfunctional or may not be expressed on CD4^+^ T_RM_ cells. In this case, however, CD103^+^ regulatory T cells contribute to the suppression of chronic inflammation mediated by CD4^+^ T_RM_ cells.

### Effector functions of T_RM_ cells on the pathology of skin diseases

For the induction of skin diseases, T_RM_ cells are strongly correlated with the outcome of disease progression. Furthermore, the functional features of T_RM_ cells differ from those of individual skin diseases [197]. Psoriasis is a chronic and recurrent autoimmune disorder mediated by IL-17A^+^IL-22^+^ T_RM_ cells, which trigger inflammation. Both CD49a^+^ and CD49a^-^CD103^+^CD8^+^ T_RM_ cells accumulate in psoriatic areas [198, 199], and increases in these T_RM_ cell subsets have also been reported in the liver of patients with autoimmune hepatitis [200]. In particular, CD49a^-^CD103^+^CD8^+^ T_RM_ cells are the major producers of proinflammatory IL-17A. In contrast, CD49a^+^CD103^+^CD8^+^ T_RM_ cells have potent cytotoxic functions in the secretion of Prf1 and GzmB. Similar to CD8^+^ T_RM_ cells, skin CD4^+^ T_RM_ cells play a role in the relapse of psoriasis in humans. IL-22, produced by CD4^+^ T_RM_ cells, affects the survival, proliferation, and differentiation of keratinocytes by producing antimicrobial proteins and chemokines [201].

In vitiligo, the skin microenvironment is completely different from that in psoriasis. Vitiligo is defined as the presence of prominent, irregular white patches on the skin triggered by CD8^+^ T cells that abnormally target melanocytes for elimination. Melanocytes in patients with vitiligo have a diminished ability to regulate cellular insults, making them more susceptible to external factors such as organic chemicals and UV exposure [202]. Therefore, a variety of immune cells are attracted to and activated by inflammatory cytokines released upon exposure to these stimuli [203]. Furthermore, the production of CXCR3 ligands mediated by IFN-γ secretion is essential for the recruitment of autoreactive CD8^+^ T cells in vitiligo in animal models and humans and is associated with the development and severity of the diseases [204–206]. Janus kinase (JAK) inhibitors blocked IFN signaling, resulting in the reversal of the disease state, while treatment with these inhibitors appeared to have no effect on the number of T_RM_ cells [207]. Therefore, future studies must examine in detail the functional differences in T_RM_ cells, including their ability to produce cytokines.

### Effector functions of T_RM_ cells on the pathology of respiratory diseases

Exposure to inhaled allergens, including a house dust mites and diesel exhaust particles, generates Th2 memory cells that contribute to airway inflammation. Among Th2 memory cells located in lung tissue, Th2-type T_RM_ cells accumulate in the lung parenchyma for a long period in mice and humans [86, 105, 208]. Th2 T_RM_ cells proliferate near the airways and induce mucus transformation, airway hyperresponsiveness, and the activation of airway eosinophils through the rapid secretion of IL-5 and IL-13. In patients with asthma, the number of CD103^+^CD4^+^ T_RM_ cells with cytotoxicity and proinflammatory cytokines increases with disease severity [78].

### Effector functions of T_RM_ cells on the pathology of intestinal diseases

IBD is a group of two diseases (including Crohn’s disease and ulcerative colitis) caused by progressive inflammation of the gastrointestinal tract. The number of CD103^+^CD4^+^ and CD103^+^CD8^+^ T_RM_ cells is increased in the gut tissues of patients with IBD [84]. In particular, the presence of CD103^+^CD4^+^ T_RM_ cells strongly correlates with the clinical relapses of IBD. Furthermore, a unique population of CD161^+^CD103^+^CD4^+^ T_RM_ cells that exert robust effector functions without TCR engagement, has been found in the intestinal tissues of patients with Crohn’s disease [209]. Depletion of T_RM_ cell precursors remarkably suppresses the inflammation caused by dextran sodium sulfate colitis in mice [30].

## The mechanism of exhibiting effector functions of T_RM_ cells following reinfection or reactivation

The presence of T_RM_ cells in peripheral tissues has a significant effect on protection against microbial reinfection and the development of autoimmune diseases, tumors, and allergic responses [37, 190]. The rapid response of T_RM_ cells to secondary infections or exposure to the same antigen has a significant impact on human wellness, as they are most effective in eliminating local infections or initiating inflammatory responses. Therefore, elucidating the mechanisms by which T_RM_ cells exert their effector functions and the mechanism by which T_RM_ cells are retained and maintained in tissues is crucial for developing new preventive and therapeutic interventions and therapeutics to control diseases that are detrimental to human health.

Two distinct pathways are needed for T_RM_ cells to exhibit their effector function. One is the pathway through which T_RM_ cells recognize antigens via TCR and then reactivate them. In the other pathway, T_RM_ cells are reactivated by receiving cytokine signals through receptors. Thus, the function of T_RM_ cells is likely to be largely influenced by environmental factors in peripheral tissues. Another important question is why a large number of T_RM_ cells localize to various tissues even though innate immune cells without TCRs also localize to many peripheral tissues that produce the same cytokines and chemokines as T_RM_ cells.

### T_RM_ cell reactivation following antigen presentation

Upon microbial infection in tissues, DCs capture antigens and upregulate costimulatory molecules and CCR7 to enter draining LNs (DLNs); in the DLNs, DCs specifically activate naïve T cells and B cells to promote their differentiation into effector cells [210]. In the case of T_M_ cells, the recall response is thought to be mediated by the direct recognition of antigens presented on MHC classes I and II upregulated on infected cells *in vitro* [211] or by CD4^+^ T-cell help *in vivo* [212]. The mechanism by which T_M_ cells are activated by antigens presented on parenchymal cells is supported by the evidence that T_M_ cells are more readily activated than T_N_ cells.

However, CD11c^+^ DCs were shown to be involved in the efficient reactivation of T_EM_ cells or T_RM_ cells following systemic microbial reinfection with *L. monocytogenes*, LCMV, and vesicular stomatitis virus (VSV) in mice [94, 213]. In contrast, the requirement for CD11c^+^ DCs in mucosal tissues for the recall response of T_RM_ cells after IAV infection is limited. In this case, both hematopoietic cell-derived APCs, including DCs and macrophages, and non-hematopoietic cells, such as epithelial and endothelial cells, present viral antigens to CD8^+^ T_RM_ cells in murine lung tissues following IAV reinfection [94], suggesting that CD8^+^ T_RM_ cells directly recognize almost all cells infected with the IAV. However, the effector mechanisms of CD8^+^ T_RM_ cells that lead to the elimination of virus-infected cells remain obscure.

Given that the secondary response of T_RM_ cells is initiated by recognition of cognate antigens presented on APCs, antigen-specific TCRs can potentially recognize multiple peptide epitopes [214]. Following infection with the primary pathogen, T cells bearing TCRs are primed against epitopes. Subsequently, some differentiated T_M_ cells can cross-react with peptides presented on APCs after the second microbe [215]. In severe acute respiratory syndrome coronavirus (SARS-CoV)-1 survivors, immunization with the SARS-CoV-2 vaccine augmented SARS-CoV-1-specific T-cell responses, suggesting the possibility of cross-reactivity. In addition to SARS-CoV-1 infection, other cases of human coronavirus infection, the virus that causes colds, especially in children during the winter season, could generate human coronavirus-specific T_RM_ cells in the lung tissue. Indeed, in SARS-CoV-2-naïve individuals, T_RM_ cells reactive to SARS-CoV-2 antigens were abundant in the lungs [216], suggesting that cross-reactive T_RM_ cells protect against SARS-CoV-2 infection.

Using the mouse model of an inducible antigen expression on keratinocytes instead of infection, skin Langerhans cells can capture the keratinocyte-derived inducible antigens and present them to CD103^+^CD8^+^ T_RM_ cells in the epidermis [217]. Thereafter, CD103^+^CD8^+^ T_RM_ cells robustly produce IFN-γ to recruit leukocytes, including inflammatory monocytes and neutrophils, to the dermal tissues. These findings suggest that the dysfunction and functional modification of APCs associated with microbial infection or the tumor microenvironment, rather than simple antigen expression in tissues, largely affect the ability of T_RM_ cells to exert effector functions.

Comparing the functions of cytotoxic activity and cytokine secretion, cytotoxic activity requires cell-to-cell interaction in a confined space [218], whereas the function of cytokine secretion is that cytokines are initially secreted by cell-to-cell interactions, but the spreading effect of cytokine secretion can widely affect the surrounding environment. For instance, IFN-γ secreted by T_E_ cells contributes to the protection against intracellular pathogens. Following TCR engagement, IFN-γ secreted from T_M_ cells acts on the infected cells. Furthermore, IFN-γ spreads from the interaction site with APCs or infected cells and reaches concentrations sufficient to activate IFN-γ signaling pathways in distant cells [219].

Considering the effector functions of T_RM_ cells, at the site of infection, such as skin and mucosal tissues, both infected cells and/or APCs present antigens to T_RM_ cells. Later, in addition to its cytotoxic activity, the rapid secretion of IFN-γ acts immediately on infected cells and diffuses from the site of infected cells and/or antigen-presenting APCs to distant infected cells to prevent dissemination to other tissues. This action of cytokines produces a wide variety of effects on non-infected cells, including the activation of innate immune cells and endothelial cells. Injection of cognate antigens into mucosal tissues in mice or mucosal viral infection activates T_RM_ cells to rapidly produce IFN-γ. This IFN-γ secretion leads to increased production of CXCR3 ligands and upregulation of vascular cell adhesion molecule 1 from CD31^+^ endothelial cells [96, 97]. Subsequently, a wide variety of immune cells, including T_M_ cells, B cells, NK cells, and APCs, infiltrate the tissues of mice [92, 93, 96]. In addition, administrating cognate antigens or recombinant IFN-γ to mucosal tissues also attracts the entry of antibodies from the circulation [95, 220].

Regarding the fate of reactivated T_RM_ cells, the majority of CD103^+^CD8^+^ T_RM_ cells proliferate massively and remain at the site of reactivation upon skin HSV infection [221], whereas the cytokine-producing capacity of CD103^+^CD8^+^ T_RM_ cells is largely inhibited by TGF-β signaling in mice [52]. In contrast, reactivated CD103^+^CD8^+^ T_RM_ cells also proliferate following LCMV challenge, but eventually leave the site of reactivation [222, 223]. On the other hand, CD103^+^CD8^+^ T_RM_ cells show the retention and limited expansion following the rechallenge with VSV or mucosal *Yersinia pseudotuberculosis* infection in mice [224, 225]. Instead, CD103^-^CD8^+^ T_RM_ cells trigger massive proliferation after *Yersinia* reinfection and have a higher cytokine-producing capacity than CD103^+^CD8^+^ T_RM_ cells [224]. Based on the fate mapping studies, CD103^-^CD8^+^ T_RM_ cells are not derived from CD103^+^CD8^+^ T_RM _cells, suggesting that CD103^+^CD8^+^ T_RM_ cells are not the memory pool for CD103^-^CD8^+^ T_RM_ cells.

The types of effector functions that T_RM_ cells can perform are determined by the nature of the invading pathogen and the characteristics of the tissue APCs. If reactivated T_RM_ cells are retained in tissues, future studies are awaited to determine how T_RM_ cells suppress effector functions after performing effector functions to eliminate pathogens in tissues, and under what conditions, such as infection, they can exhibit effector functions. In addition, the protective function of CD103^+^ T_RM_ cells in the epithelium against infection, tumor development, and autoimmune diseases, other than the functional capacity they exert, such as cytokine secretion, compared with the role of CD103^- ^T_RM_ cells in the lamina propria against microbial infection [225], is a subject for future research.

### T_RM_ cell reactivation independent of antigen presentation

In numerous experimental settings of microbial infection, T_RM_ cells at the site of infection are undoubtedly necessary for rapid and complete protection against reinfection compared with circulating T_M_ cells [29, 91, 108, 139, 143, 155]. For T_RM_ cells to perform their effector function after microbial rechallenge, they must be activated by antigen-specific TCRs to eliminate invading pathogens. T_RM_ cells also contribute to the prevention of invasion by unrelated microorganisms; however, T_RM_ cell activation via recognition of cognate antigens prior to infection is essential [226].

In addition to TCR-mediated activation of T_RM_ cells, TCR-independent activation of T_RM_ cells has also been demonstrated in some situations in mice and humans. Both CD103^+^ and CD103^-^CD8^+^ T_RM_ cells in the lung tissue produce IFN-γ following the inhalation of unrelated bacterial antigens (*in vivo* and *in vitro*) or the addition of recombinant IL-12 and IL-18 (*in vitro*) [227]. Furthermore, CD8^+^ T_RM_ cells contribute to the inhibition of the bacterial load in lung tissue in a TCR-independent manner [227], whereas the relative contribution of the effector function of TCR-unrelated CD8^+^ T_RM_ cells compared with that of antigen-specific CD8^+^ T_RM_ cells remains unclear. Similar to CD8^+^ T_RM_ cells in the lung tissue, helminth-specific CD4^+^ T_RM_ cells in the peritoneal cavity, but not in the small intestine of mice, can produce IL-5 and IL-13 upon treatment with IL-33 and IL-7 in a TCR-independent manner [159]. In this case, adoptively transferred CD4^+^ T_RM_ cells from the peritoneal cavity reduced helminth fecundity; however, the involvement of the TCR-independent function of CD4^+^ T_RM_ cells remains obscure.

In addition to the protective function of T_RM_ cells against infectious agents, T_RM_ cells in peripheral tissues exert effector functions in a TCR-independent manner under pathological conditions. In human skin, CD49a^+^CD103^+^CD8^+^ T_RM_ cells have higher cytotoxic activity by secreting Prf1 and GzmB without TCR engagement following treatment with IL-2 or IL-15 than CD49a^-^CD103^+^CD8^+^ T_RM_ cells [199]. Furthermore, TCR-mediated stimulation has an additive effect on the cytotoxic function of T_RM_ cells after IL-15 treatment, suggesting that both TCR-mediated and TCR-independent stimulation of T_RM_ cells exacerbate the pathogenesis of skin diseases.

The frequency of CD49a^+^CD103^+^CD8^+^ T_RM_ cells secreting Prf1 and GzmB was increased in the vitiligo lesions of patients, suggesting that the production of IL-2 or IL-15 is enriched in the environment of vitiligo areas. In contrast, the cytotoxicity of CD49a^+^CD103^+^CD8^+^ T_RM_ cells was relatively inferior to that of CD49a^-^CD103^+^CD8^+^ T_RM_ cells in the skin tissues of patients with psoriasis. In terms of cytokine production, both CD49a^+^ and CD49a^-^CD103^+^CD8^+^ T_RM_ cells produced high levels of IFN-γ but not IL-17A in vitiligo patients, whereas CD49a^+^ and CD49a^-^CD103^+^CD8^+^ T_RM_ cells produced high levels of IFN-γ and IL-17A, respectively, in patients with psoriasis [199, 201]. The secretion of IFN-γ and IL-17A by CD103^+^CD8^+^ T_RM_ cells requires TCR engagement.

JAK inhibitors have been shown to restore vitiligo in mice without affecting the number of T_RM_ cells [207]. This finding suggests that cytokine-activated T_RM_ cells cause the pathogenesis of vitiligo; as in patients with alopecia areata, overproduction of IL-2 or IL-15 by non-T_RM_ cells somehow activates NKG2D expression on CD103^+^CD8^+^ T_RM_-like cells to secrete Prf1 and GzmB in a TCR-independent manner [228]. Subsequently, the activated antigen-specific CD103^+^CD8^+^ T_RM_ cells destroy melanocytes via TCR-dependent cytotoxic activity (Fig. 2).

In the intestinal tissues of patients with IBD, CD4^+^ T_RM_ cells drive the clinical relapses of IBD, including Crohn’s disease and ulcerative colitis [30]. In particular, CD161^+^CD103^+^CD4^+^ T_RM_ cells secrete IFN-γ in a TCR-independent manner following treatment with IL-7, IL-15, IL-12, and IL-18 [209]. IFN-γ produced by activated CD4^+^ T_RM_ cells plays a pivotal role in the induction of epithelial cell death in humans. In addition to CD161^+^CD103^+^CD4^+^ T_RM_ cells, CD127^+^ ILC1 cells are observed to accumulate in the damaged intestinal tissue of patients with Crohn’s disease. Non-cytolytic CD127^+^ ILC1 cells can produce IFN-γ following treatment with IL-12 and IL-18 [229], whereas NK cells fail to exert effector functions, including IFN-γ secretion and cytotoxic activity, in patients with Crohn’s disease [230]. Therefore, the TCR-independent mechanism of IFN-γ-mediated cytotoxicity by T_RM_ cells and ILC1 cells contributes to the pathogenesis of IBD, whereas the division of roles in pathogenesis by these cells and the involvement of TCR-dependent cytotoxic granules by T_RM_ cells remain unclear.

Regarding the effector functions of antigen-specific T_M_ cells against unrelated antigens, the existence of memory phenotype T cells independent of previous pathogen exposure, “virtual memory T cells; T_VM_”, is emerging in mice and humans [231, 232, 233]. T_N_ cells are likely to develop into CD44^hi^CD62L^+^ T_VM_ cells in line with the expression of CD5 in mice [234]. Furthermore, IL-15 is required to develop and protect T_VM_ cells against the infections caused by unrelated pathogens. In humans, CD45RA^+^Eomes^+^KIR^+^NKG2A^+^ T cells produce IFN-γ in response to IL-12 and IL-18 [234]. Furthermore, a similar subset of T_VM_ cells has been detected in the skin of patients with alopecia areata [228].

**Figure 1.**
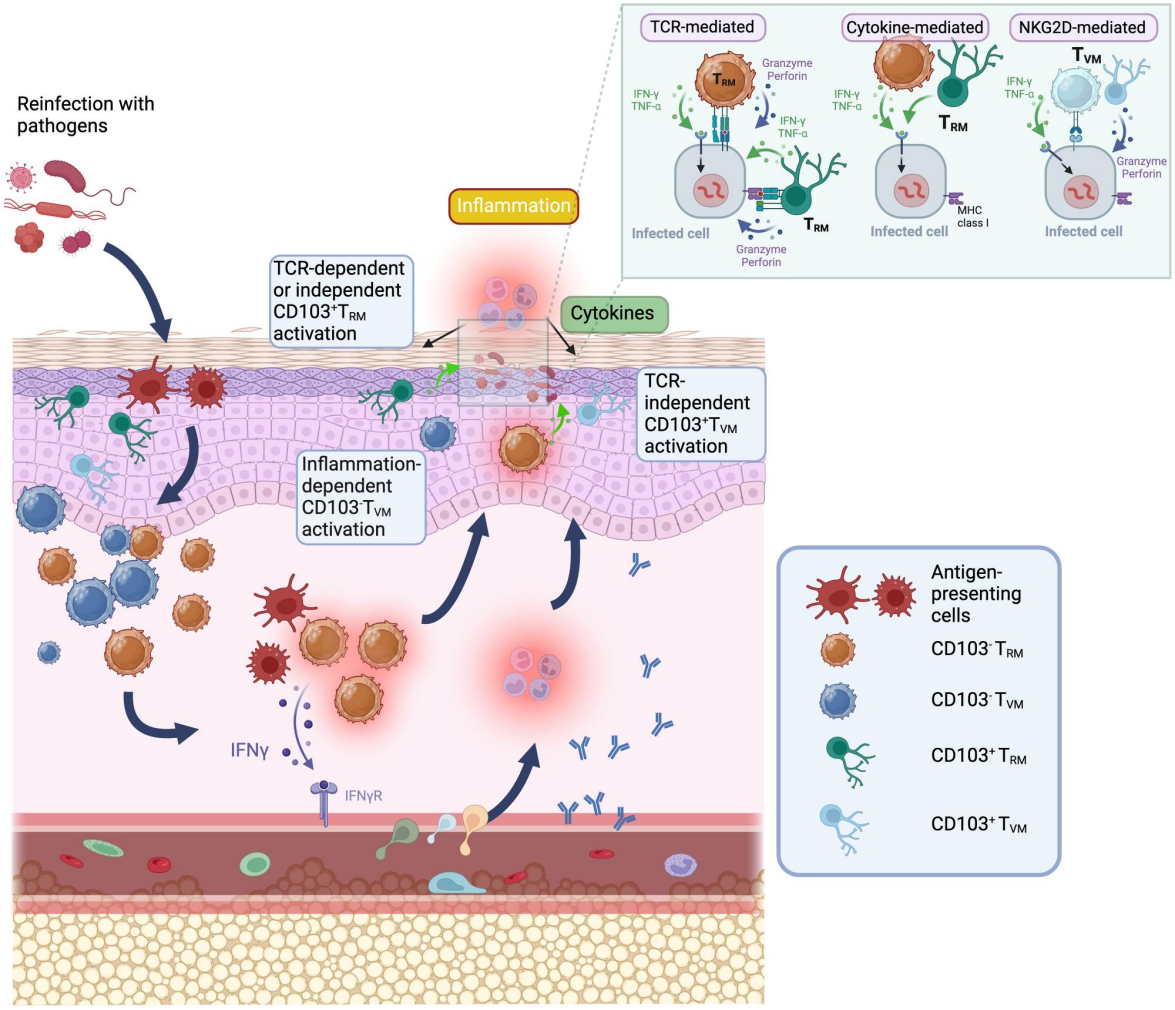
Potential mechanism of exerting effector functions mediated by T_RM_ and T_VM_ cells under pathogen reinfection. Design with BioRender (https://www.biorender.com).

In contrast to T_N_ cells, T_VM_ cells are a heterogeneous population that express CXCR3, CXCR5, CCR5, and CCR2. Within the T_VM_ cell population, both CCR2^+^ T_VM_ and CCR2^- ^T_VM_ cells infiltrate the lung tissue of mice within one day of IAV infection [235]. Moreover, CCR2^+^ T_VM_ cells show superior protection against unrelated bacterial infections compared with CCR2^- ^T_VM_ cells and T_N_ cells, but the mechanism by which CCR2^+^ T_VM_ cells act directly or helps other types of immune cells remains unclear (Fig. 1). In contrast to the effector function of CCR2^+^ T_VM_ cells in the early phase of infection, CCR2^- ^T_VM_ cells have a higher capacity to differentiate into CD103^+^ CD69^+ ^T_RM_ cells in the lung tissue than T_N_ cells. The mechanism by which CCR2^- ^T_VM_-derived T_RM_ cells exert their effector function against infection, the tumor microenvironment, and inflammatory conditions remains unclear (Fig. 1).

## Conclusion

In healthy individuals, T_RM_ cells that develop in peripheral tissues are an important subset responsible for immune surveillance by recognizing cognate antigens presented to MHC molecules without error in the peripheral tissues, which is the first line of defense. This sophisticated regulation of the antigen recognition mechanism is probably why immunosuppression and excessive inflammation do not occur. However, when excessive inflammation or chronic diseases caused by pathogens, autoimmune responses, or allergic reactions develop in peripheral tissues, the widespread release of various inflammatory molecules and cytokines into the tissue microenvironment is likely to significantly affect T_RM_ cell function by receiving their signals.

**Figure 2.**
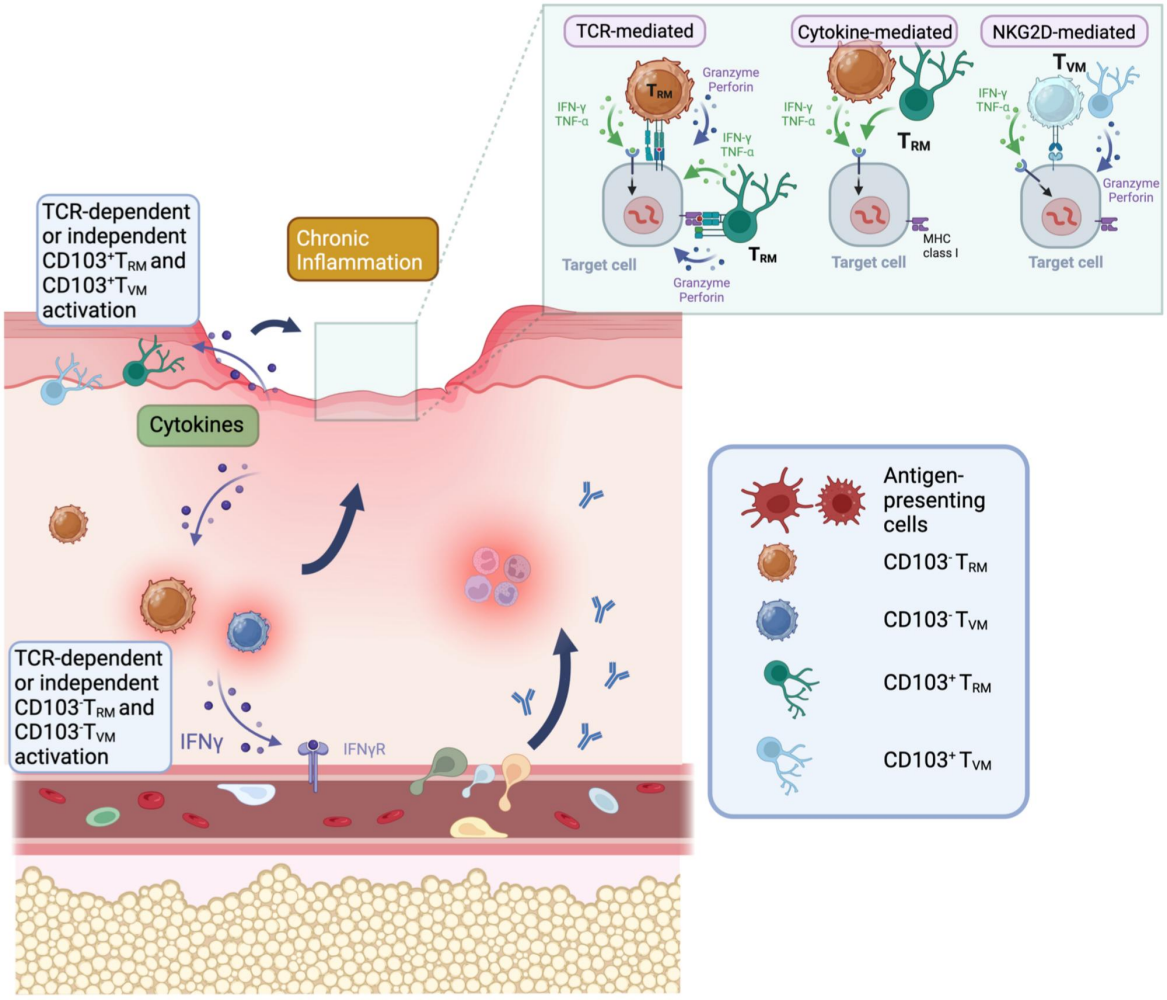
Potential mechanism of exerting effector functions mediated by T_RM_ and T_VM_ cells under pathological conditions. Design with BioRender (https://www.biorender.com).

As representative examples of TCR-independent reactivation of T cells, CD49a^+^CD8^+^ T_RM_ cells in the skin tissue of patients with vitiligo, CD161^+^CD103^+^CD4^+^ T_RM_ cells in the intestinal tissue of patients with Crohn’s disease, and KIR^+^NKG2A^+^NKG2D^+^CD8^+^ T_VM_ cells in patients with alopecia areata can exert cytotoxic functions, including the secretion of Prf1 and Gzms, and the production of IFN-γ [199, 209, 228]. Subsequent antigen-specific reactivation of T_RM_ cells may amplify secondary immune responses. These pathogenic T cells mimic NK-like behavior. However, dysregulated T cells appear to target non-specific cells expressing activating ligands such as NKG2D and NKG2A and cytokine receptors such as TNFR and IFN-γR regardless of MHC class I expression, distinct from NK cells, which target only cells expressing activating ligands with MHC class I downregulation (Fig. 2). Similar pathological conditions have been observed in the gut tissues of patients with celiac disease. Activation status of gluten-specific CD4^+^ T cells in the lamina propria and cytolytic CD8^+^ intestinal intraepithelial lymphocytes (IELs) are associated with the pathogenesis of this disease. Although the tissue residency of these pathogenic T cells remains obscure, CD8^+^ IELs exert cytolytic functions similar to those of NK cells in a TCR-independent manner in humans [236, 237].

The homeostatic microenvironment of peripheral tissues should be maintained for T_RM_ cells to function properly. Once the homeostasis of the microenvironment is disrupted, not only are antigen non-specific T cells such as T_VM_ cells activated, but T_RM_ cells are also activated in a non-specific manner. Additionally, if T_RM_ cells respond to cognate antigens, the disease status may be further exacerbated (Fig. 2).

Contrary to the role of T_RM_ cells in pathological conditions, T_RM_ cells are a pivotal element of the immune defense of peripheral tissues against invading pathogens [37, 238, 239]. In addition, considering the functional properties of T_VM_ cells following immunization to target the infiltration of T_RM_ cells, the accumulation of T_VM_ cells may help combat the spread of microbes (Fig.1). In the case of MLC formed in the genital tissues of mice following attenuated HSV-2 immunization, approximately 30% of T_RM_ cells express TCRVβ1, which can produce IFN-γ in an antigen-specific manner [91], whereas the phenotype of the remaining T_RM_ cells is largely unknown. Similarly, MLC-like formation has also been detected in the intestinal tissue of mice following *Y. pseudotuberculosis* infection [122]. Therefore, MLCs that develop in the genital or intestinal mucosa may also harbor tissue-resident T_VM_ cells [225].

In contrast, the activation of tissue-resident T_VM_ cells may create a pro-inflammatory state in the tissue microenvironment following pathogen invasion (Fig. 2). Subsequently, these T_VM_ cell subsets may terminate the silent status of autoantigen-specific T cells and induce their activation, thereby allowing these T cells to cause tissue damage. The activation of tissue-resident T_VM_ cells could explain one of the mechanisms by which microbial infections influences the development of autoimmune diseases.

## Data Availability

There is no data associated with this review.
